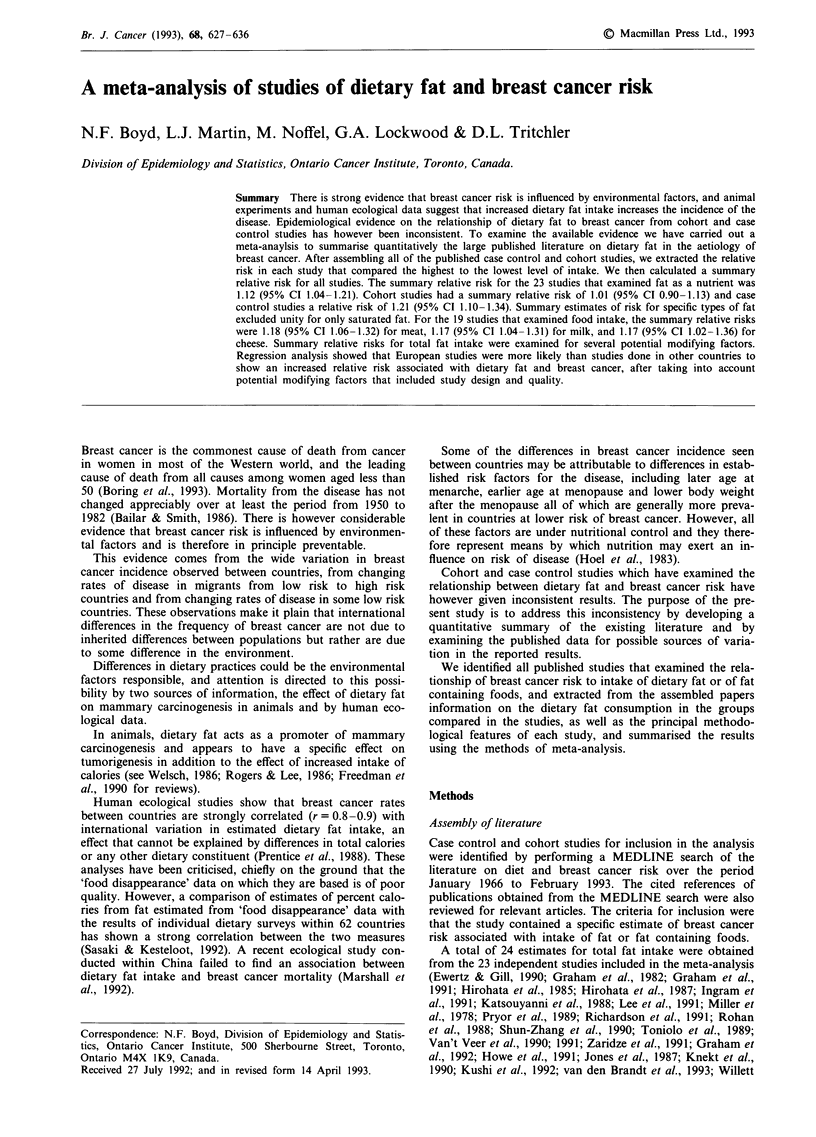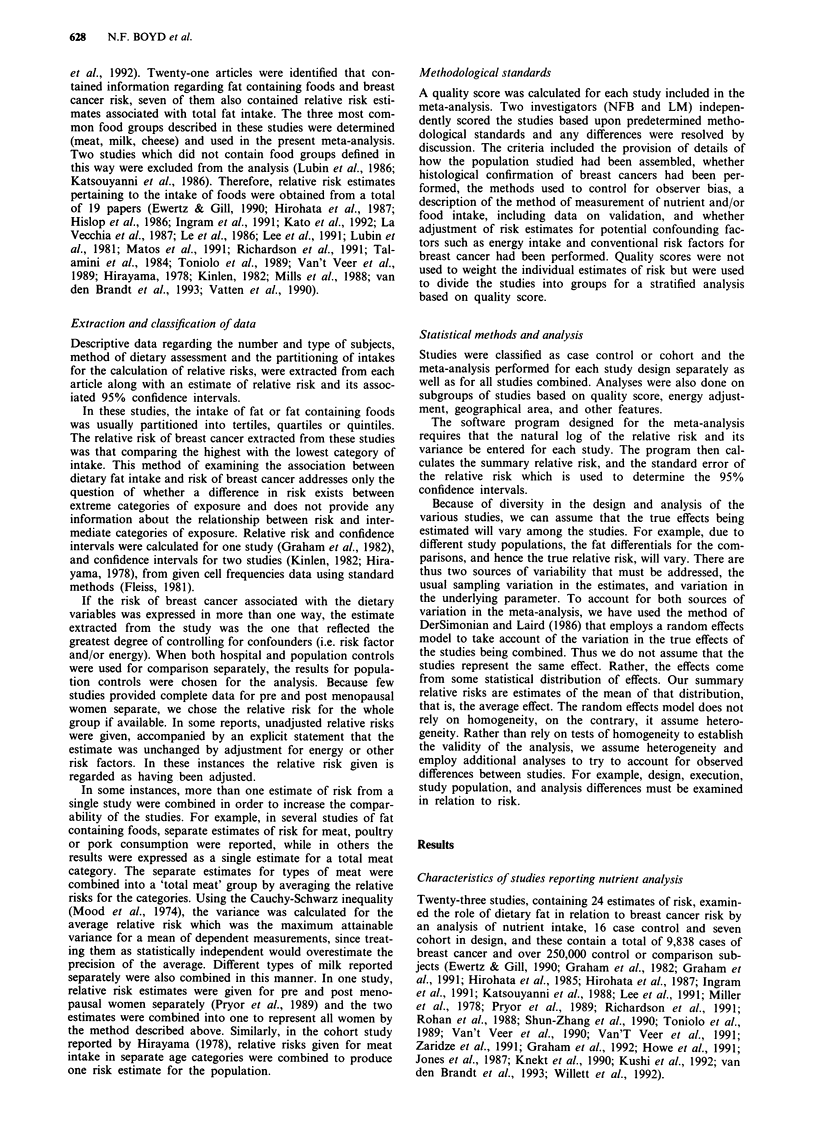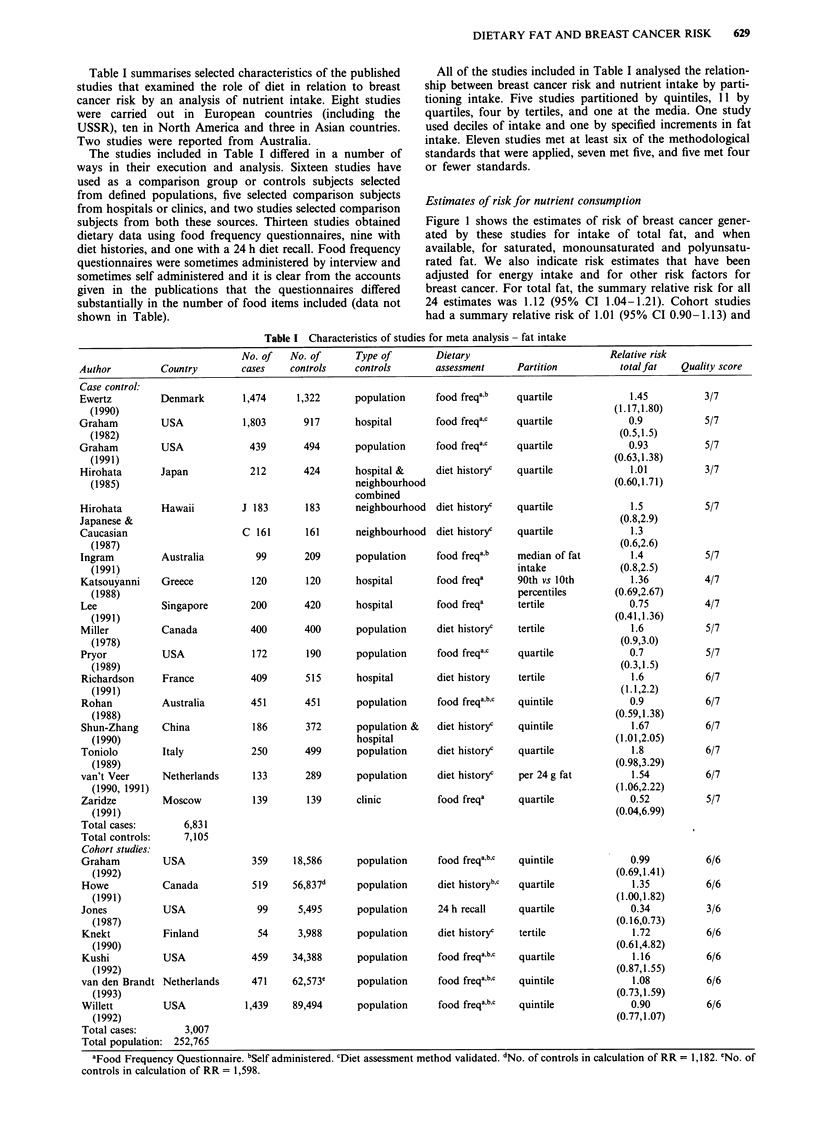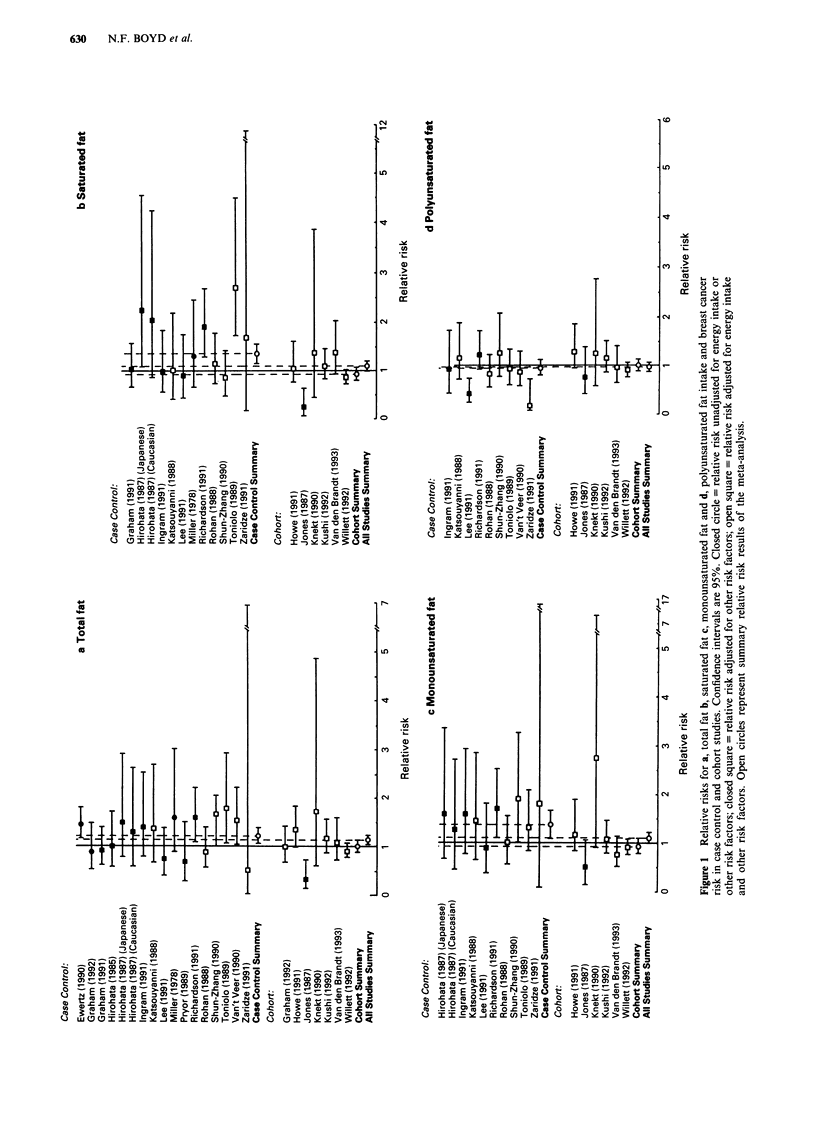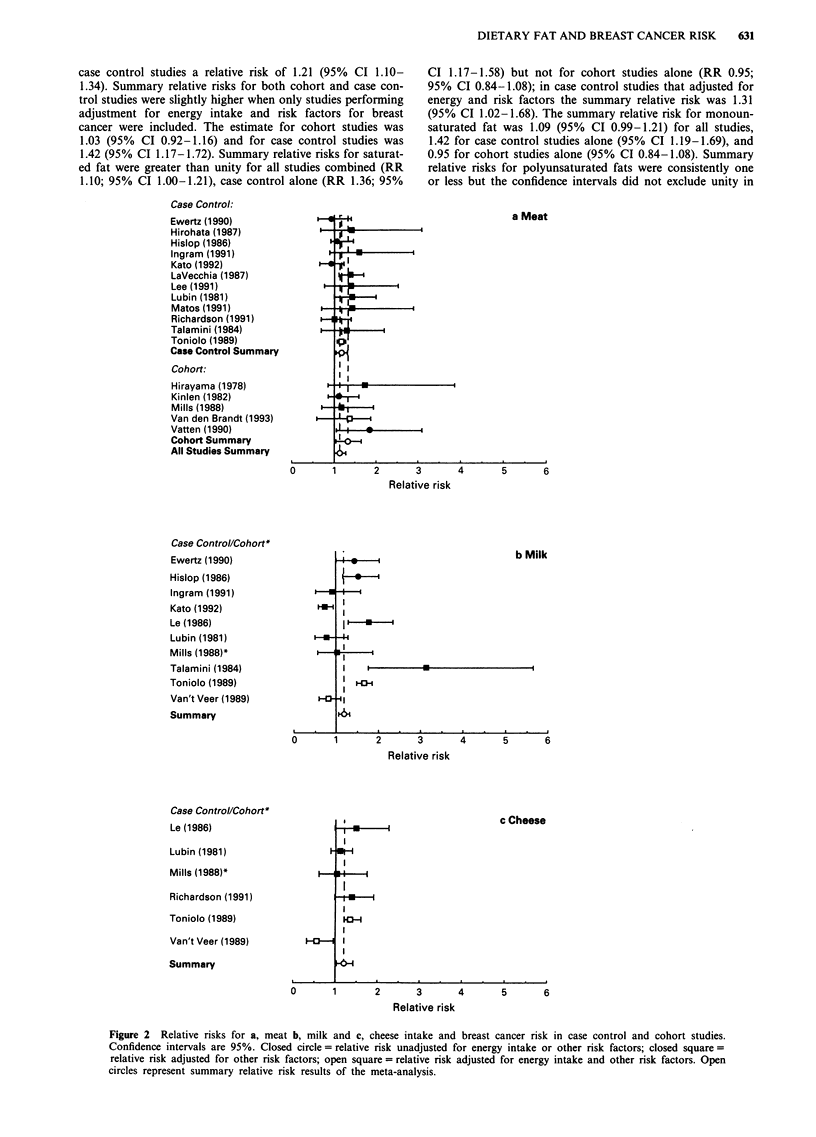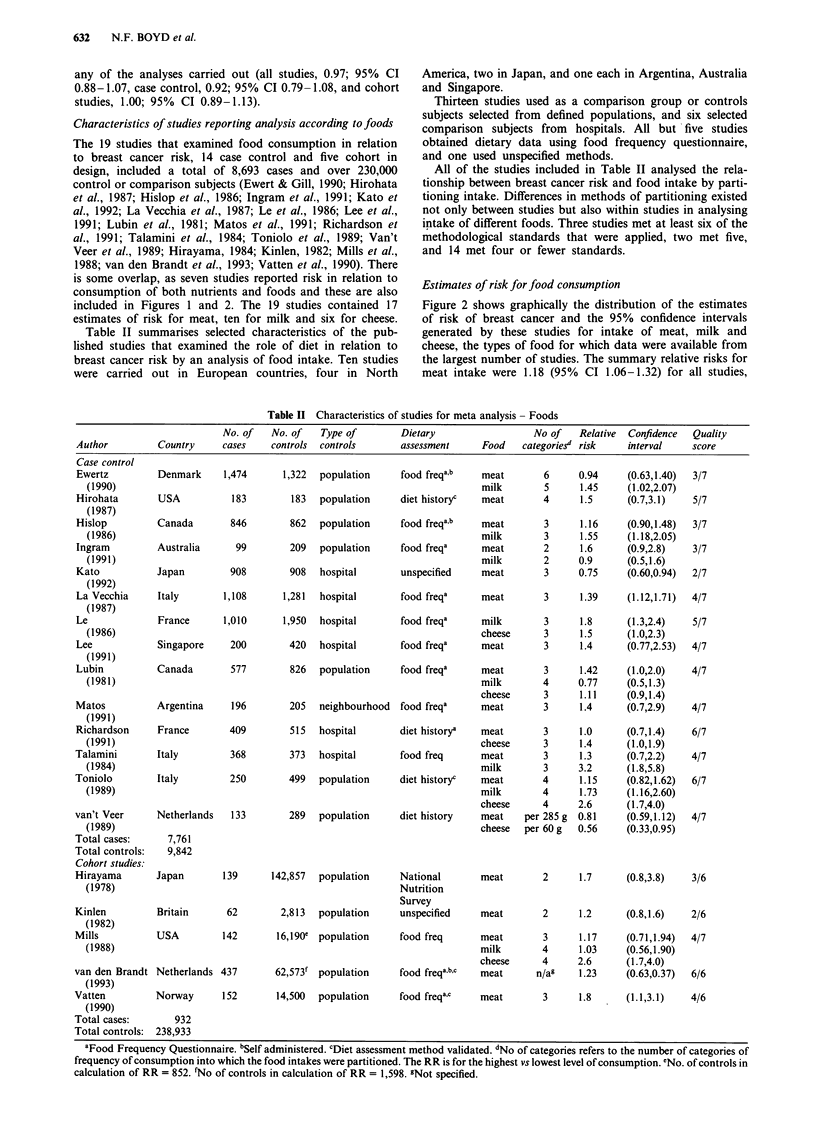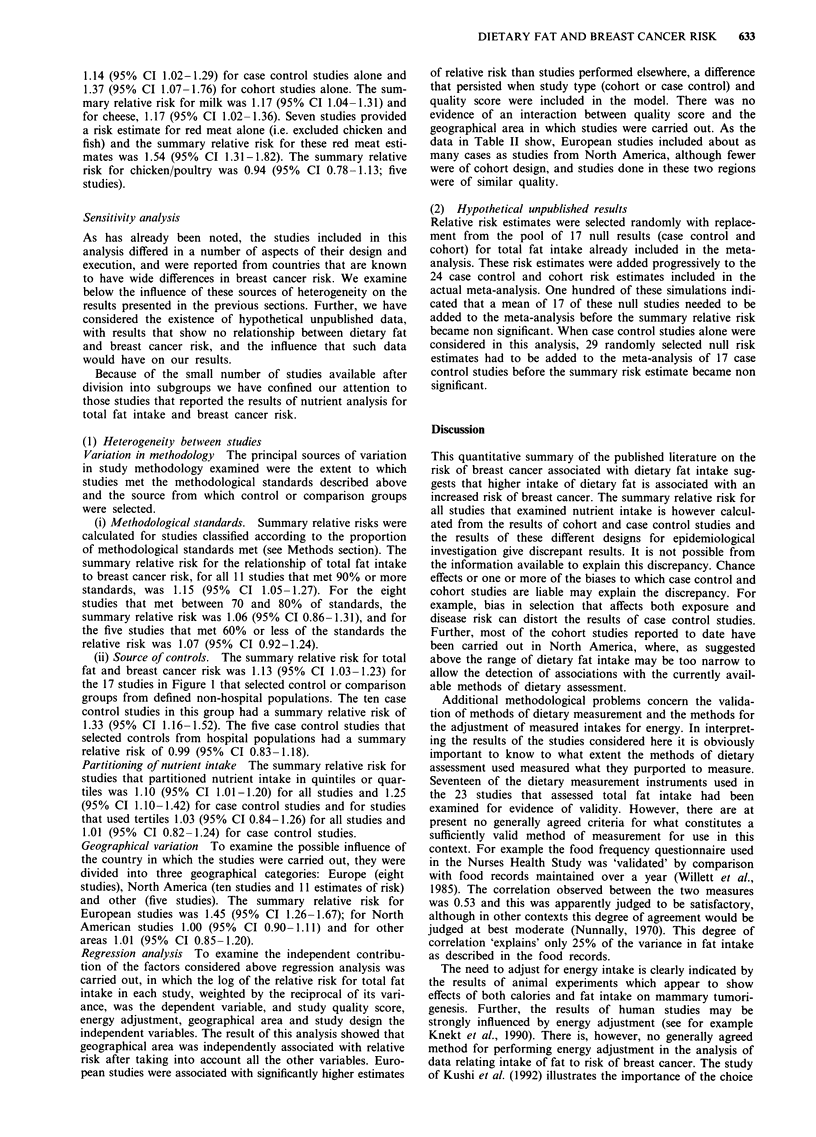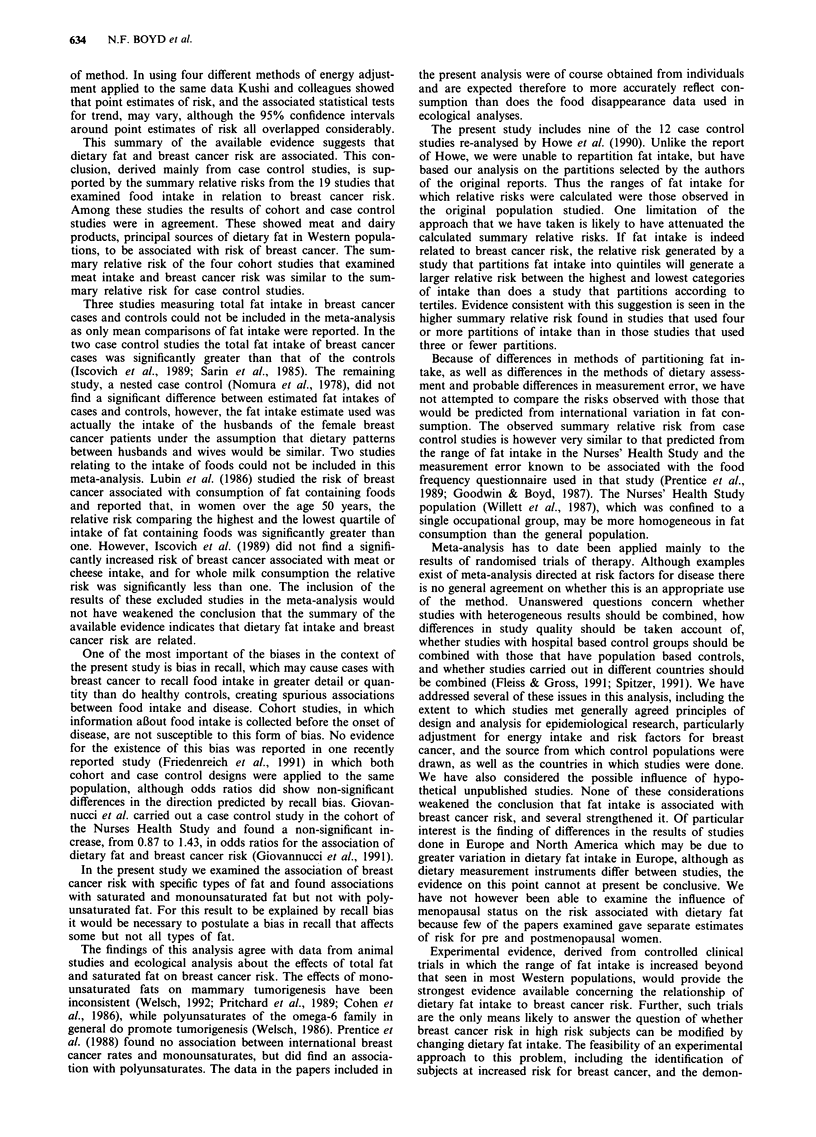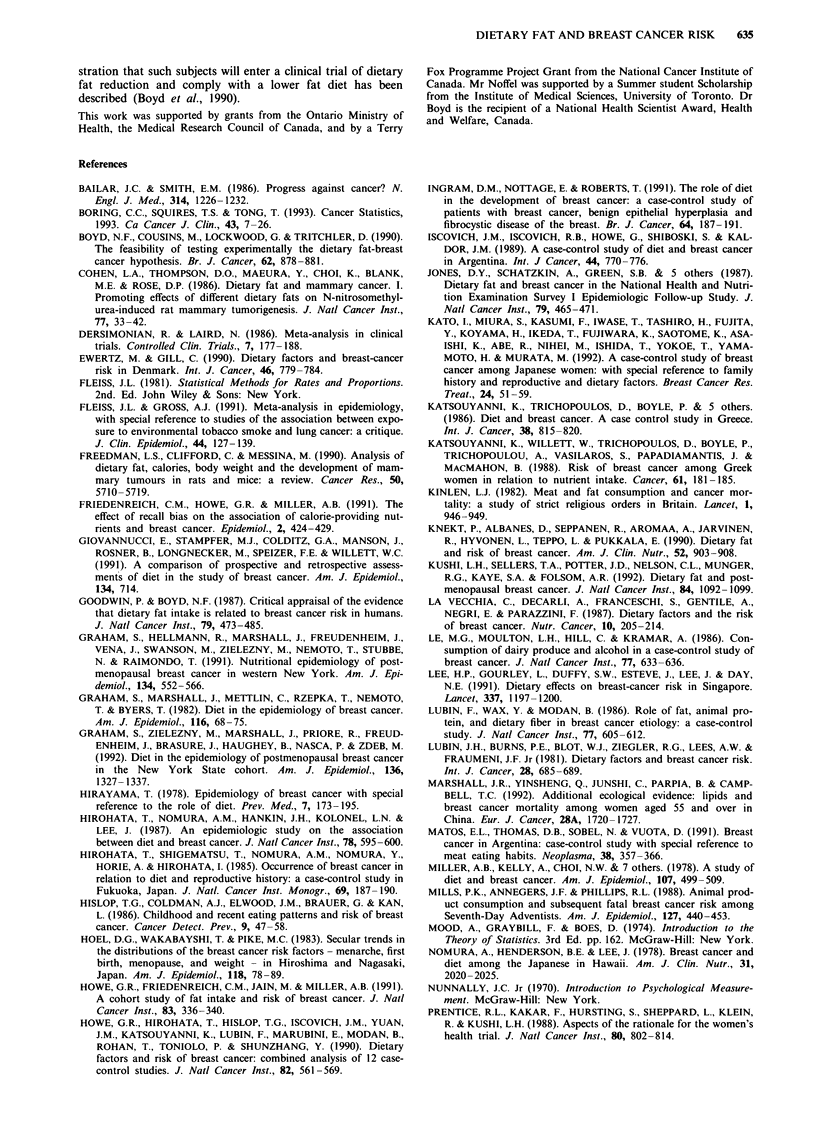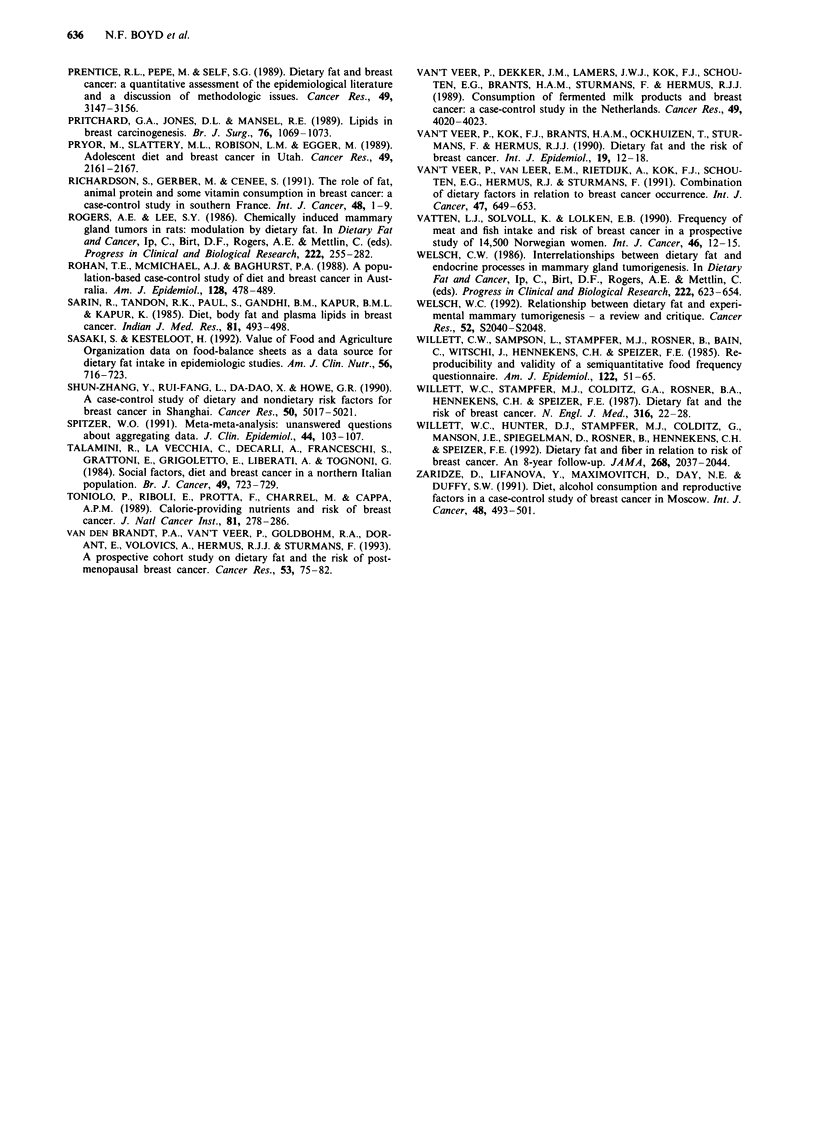# A meta-analysis of studies of dietary fat and breast cancer risk.

**DOI:** 10.1038/bjc.1993.398

**Published:** 1993-09

**Authors:** N. F. Boyd, L. J. Martin, M. Noffel, G. A. Lockwood, D. L. Trichler

**Affiliations:** Division of Epidemiology and Statistics, Ontario Cancer Institute, Toronto, Canada.

## Abstract

There is strong evidence that breast cancer risk is influenced by environmental factors, and animal experiments and human ecological data suggest that increased dietary fat intake increases the incidence of the disease. Epidemiological evidence on the relationship of dietary fat to breast cancer from cohort and case control studies has however been inconsistent. To examine the available evidence we have carried out a meta-analysis to summarise quantitatively the large published literature on dietary fat in the aetiology of breast cancer. After assembling all of the published case control and cohort studies, we extracted the relative risk in each study that compared the highest to the lowest level of intake. We then calculated a summary relative risk for all studies. The summary relative risk for the 23 studies that examined fat as a nutrient was 1.12 (95% CI 1.04-1.21). Cohort studies had a summary relative risk of 1.01 (95% CI 0.90-1.13) and case control studies a relative risk of 1.21 (95% CI 1.10-1.34). Summary estimates of risk for specific types of fat excluded unity for only saturated fat. For the 19 studies that examined food intake, the summary relative risks were 1.18 (95% CI 1.06-1.32) for meat, 1.17 (95% CI 1.04-1.31) for milk, and 1.17 (95% CI 1.02-1.36) for cheese. Summary relative risks for total fat intake were examined for several potential modifying factors. Regression analysis showed that European studies were more likely than studies done in other countries to show an increased relative risk associated with dietary fat and breast cancer, after taking into account potential modifying factors that included study design and quality.


*Br. J. Cancer* (1993), **68**, 627–636 © Macmillan Press Ltd., 1993

### A meta-analysis of studies of dietary fat and breast cancer risk

N.F. Boyd, L.J. Martin, M. Noffel, G.A. Lockwood & D.L. Tritchler

*Division of Epidemiology and Statistics, Ontario Cancer Institute, Toronto, Canada.*

**Summary** There is strong evidence that breast cancer risk is influenced by environmental factors, and animal experiments and human ecological data suggest that increased dietary fat intake increases the incidence of the disease. Epidemiological evidence on the relationship of dietary fat to breast cancer from cohort and case control studies has however been inconsistent. To examine the available evidence we have carried out a meta-anaylsis to summarise quantitatively the large published literature on dietary fat in the aetiology of breast cancer. After assembling all of the published case control and cohort studies, we extracted the relative risk in each study that compared the highest to the lowest level of intake. We then calculated a summary relative risk for all studies. The summary relative risk for the 23 studies that examined fat as a nutrient was 1.12 (95% CI 1.04–1.21). Cohort studies had a summary relative risk of 1.01 (95% CI 0.90–1.13) and case control studies a relative risk of 1.21 (95% CI 1.10–1.34). Summary estimates of risk for specific types of fat excluded unity for only saturated fat. For the 19 studies that examined food intake, the summary relative risks were 1.18 (95% CI 1.06–1.32) for meat, 1.17 (95% CI 1.04–1.31) for milk, and 1.17 (95% CI 1.02–1.36) for cheese. Summary relative risks for total fat intake were examined for several potential modifying factors. Regression analysis showed that European studies were more likely than studies done in other countries to show an increased relative risk associated with dietary fat and breast cancer, after taking into account potential modifying factors that included study design and quality.

Breast cancer is the commonest cause of death from cancer in women in most of the Western world, and the leading cause of death from all causes among women aged less than 50 (Boring *et al.*, 1993). Mortality from the disease has not changed appreciably over at least the period from 1950 to 1982 (Bailar & Smith, 1986). There is however considerable evidence that breast cancer risk is influenced by environmental factors and is therefore in principle preventable.

This evidence comes from the wide variation in breast cancer incidence observed between countries, from changing rates of disease in migrants from low risk to high risk countries and from changing rates of disease in some low risk countries. These observations make it plain that international differences in the frequency of breast cancer are not due to inherited differences between populations but rather are due to some difference in the environment.

Differences in dietary practices could be the environmental factors responsible, and attention is directed to this possibility by two sources of information, the effect of dietary fat on mammary carcinogenesis in animals and by human ecological data.

In animals, dietary fat acts as a promoter of mammary carcinogenesis and appears to have a specific effect on tumorigenesis in addition to the effect of increased intake of calories (see Welsch, 1986; Rogers & Lee, 1986; Freedman *et al.*, 1990 for reviews).

Human ecological studies show that breast cancer rates between countries are strongly correlated ($r = 0.8–0.9$) with international variation in estimated dietary fat intake, an effect that cannot be explained by differences in total calories or any other dietary constituent (Prentice *et al.*, 1988). These analyses have been criticised, chiefly on the ground that the 'food disappearance' data on which they are based is of poor quality. However, a comparison of estimates of percent calories from fat estimated from 'food disappearance' data with the results of individual dietary surveys within 62 countries has shown a strong correlation between the two measures (Sasaki & Kesteloot, 1992). A recent ecological study conducted within China failed to find an association between dietary fat intake and breast cancer mortality (Marshall *et al.*, 1992).

Correspondence: N.F. Boyd, Division of Epidemiology and Statistics, Ontario Cancer Institute, 500 Sherbourne Street, Toronto, Ontario M4X 1K9, Canada.
Received 27 July 1992; and in revised form 14 April 1993.

Some of the differences in breast cancer incidence seen between countries may be attributable to differences in established risk factors for the disease, including later age at menarche, earlier age at menopause and lower body weight after the menopause all of which are generally more prevalent in countries at lower risk of breast cancer. However, all of these factors are under nutritional control and they therefore represent means by which nutrition may exert an influence on risk of disease (Hoel *et al.*, 1983).

Cohort and case control studies which have examined the relationship between dietary fat and breast cancer risk have however given inconsistent results. The purpose of the present study is to address this inconsistency by developing a quantitative summary of the existing literature and by examining the published data for possible sources of variation in the reported results.

We identified all published studies that examined the relationship of breast cancer risk to intake of dietary fat or of fat containing foods, and extracted from the assembled papers information on the dietary fat consumption in the groups compared in the studies, as well as the principal methodological features of each study, and summarised the results using the methods of meta-analysis.

### Methods

### *Assembly of literature*

Case control and cohort studies for inclusion in the analysis were identified by performing a MEDLINE search of the literature on diet and breast cancer risk over the period January 1966 to February 1993. The cited references of publications obtained from the MEDLINE search were also reviewed for relevant articles. The criteria for inclusion were that the study contained a specific estimate of breast cancer risk associated with intake of fat or fat containing foods.

A total of 24 estimates for total fat intake were obtained from the 23 independent studies included in the meta-analysis (Ewertz & Gill, 1990; Graham *et al.*, 1982; Graham *et al.*, 1991; Hirohata *et al.*, 1985; Hirohata *et al.*, 1987; Ingram *et al.*, 1991; Katsouyanni *et al.*, 1988; Lee *et al.*, 1991; Miller *et al.*, 1978; Pryor *et al.*, 1989; Richardson *et al.*, 1991; Rohan *et al.*, 1988; Shun-Zhang *et al.*, 1990; Toniolo *et al.*, 1989; Van't Veer *et al.*, 1990; 1991; Zaridze *et al.*, 1991; Graham *et al.*, 1992; Howe *et al.*, 1991; Jones *et al.*, 1987; Knekt *et al.*, 1990; Kushi *et al.*, 1992; van den Brandt *et al.*, 1993; Willett et al., 1992). Twenty-one articles were identified that contained information regarding fat containing foods and breast cancer risk, seven of them also contained relative risk estimates associated with total fat intake. The three most common food groups described in these studies were determined (meat, milk, cheese) and used in the present meta-analysis. Two studies which did not contain food groups defined in this way were excluded from the analysis (Lubin et al., 1986; Katsouyanni et al., 1986). Therefore, relative risk estimates pertaining to the intake of foods were obtained from a total of 19 papers (Ewertz & Gill, 1990; Hirohata et al., 1987; Hislop et al., 1986; Ingram et al., 1991; Kato et al., 1992; La Vecchia et al., 1987; Le et al., 1986; Lee et al., 1991; Lubin et al., 1981; Matos et al., 1991; Richardson et al., 1991; Talamini et al., 1984; Toniolo et al., 1989; Van't Veer et al., 1989; Hirayama, 1978; Kinlen, 1982; Mills et al., 1988; van den Brandt et al., 1993; Vatten et al., 1990).

*Extraction and classification of data*

Descriptive data regarding the number and type of subjects, method of dietary assessment and the partitioning of intakes for the calculation of relative risks, were extracted from each article along with an estimate of relative risk and its associated 95% confidence intervals.

In these studies, the intake of fat or fat containing foods was usually partitioned into tertiles, quartiles or quintiles. The relative risk of breast cancer extracted from these studies was that comparing the highest with the lowest category of intake. This method of examining the association between dietary fat intake and risk of breast cancer addresses only the question of whether a difference in risk exists between extreme categories of exposure and does not provide any information about the relationship between risk and intermediate categories of exposure. Relative risk and confidence intervals were calculated for one study (Graham et al., 1982), and confidence intervals for two studies (Kinlen, 1982; Hirayama, 1978), from given cell frequencies data using standard methods (Fleiss, 1981).

If the risk of breast cancer associated with the dietary variables was expressed in more than one way, the estimate extracted from the study was the one that reflected the greatest degree of controlling for confounders (i.e. risk factor and/or energy). When both hospital and population controls were used for comparison separately, the results for population controls were chosen for the analysis. Because few studies provided complete data for pre and post menopausal women separate, we chose the relative risk for the whole group if available. In some reports, unadjusted relative risks were given, accompanied by an explicit statement that the estimate was unchanged by adjustment for energy or other risk factors. In these instances the relative risk given is regarded as having been adjusted.

In some instances, more than one estimate of risk from a single study were combined in order to increase the comparability of the studies. For example, in several studies of fat containing foods, separate estimates of risk for meat, poultry or pork consumption were reported, while in others the results were expressed as a single estimate for a total meat category. The separate estimates for types of meat were combined into a 'total meat' group by averaging the relative risks for the categories. Using the Cauchy-Schwarz inequality (Mood et al., 1974), the variance was calculated for the average relative risk which was the maximum attainable variance for a mean of dependent measurements, since treating them as statistically independent would overestimate the precision of the average. Different types of milk reported separately were also combined in this manner. In one study, relative risk estimates were given for pre and post menopausal women separately (Pryor et al., 1989) and the two estimates were combined into one to represent all women by the method described above. Similarly, in the cohort study reported by Hirayama (1978), relative risks given for meat intake in separate age categories were combined to produce one risk estimate for the population.

*Methodological standards*

A quality score was calculated for each study included in the meta-analysis. Two investigators (NFB and LM) independently scored the studies based upon predetermined methodological standards and any differences were resolved by discussion. The criteria included the provision of details of how the population studied had been assembled, whether histological confirmation of breast cancers had been performed, the methods used to control for observer bias, a description of the method of measurement of nutrient and/or food intake, including data on validation, and whether adjustment of risk estimates for potential confounding factors such as energy intake and conventional risk factors for breast cancer had been performed. Quality scores were not used to weight the individual estimates of risk but were used to divide the studies into groups for a stratified analysis based on quality score.

*Statistical methods and analysis*

Studies were classified as case control or cohort and the meta-analysis performed for each study design separately as well as for all studies combined. Analyses were also done on subgroups of studies based on quality score, energy adjustment, geographical area, and other features.

The software program designed for the meta-analysis requires that the natural log of the relative risk and its variance be entered for each study. The program then calculates the summary relative risk, and the standard error of the relative risk which is used to determine the 95% confidence intervals.

Because of diversity in the design and analysis of the various studies, we can assume that the true effects being estimated will vary among the studies. For example, due to different study populations, the fat differentials for the comparisons, and hence the true relative risk, will vary. There are thus two sources of variability that must be addressed, the usual sampling variation in the estimates, and variation in the underlying parameter. To account for both sources of variation in the meta-analysis, we have used the method of DerSimonian and Laird (1986) that employs a random effects model to take account of the variation in the true effects of the studies being combined. Thus we do not assume that the studies represent the same effect. Rather, the effects come from some statistical distribution of effects. Our summary relative risks are estimates of the mean of that distribution, that is, the average effect. The random effects model does not rely on homogeneity, on the contrary, it assume heterogeneity. Rather than rely on tests of homogeneity to establish the validity of the analysis, we assume heterogeneity and employ additional analyses to try to account for observed differences between studies. For example, design, execution, study population, and analysis differences must be examined in relation to risk.

### Results

*Characteristics of studies reporting nutrient analysis*

Twenty-three studies, containing 24 estimates of risk, examined the role of dietary fat in relation to breast cancer risk by an analysis of nutrient intake, 16 case control and seven cohort in design, and these contain a total of 9,838 cases of breast cancer and over 250,000 control or comparison subjects (Ewertz & Gill, 1990; Graham et al., 1982; Graham et al., 1991; Hirohata et al., 1985; Hirohata et al., 1987; Ingram et al., 1991; Katsouyanni et al., 1988; Lee et al., 1991; Miller et al., 1978; Pryor et al., 1989; Richardson et al., 1991; Rohan et al., 1988; Shun-Zhang et al., 1990; Toniolo et al., 1989; Van't Veer et al., 1990; Van'T Veer et al., 1991; Zaridze et al., 1991; Graham et al., 1992; Howe et al., 1991; Jones et al., 1987; Knekt et al., 1990; Kushi et al., 1992; van den Brandt et al., 1993; Willett et al., 1992).

Table I summarises selected characteristics of the published studies that examined the role of diet in relation to breast cancer risk by an analysis of nutrient intake. Eight studies were carried out in European countries (including the USSR), ten in North America and three in Asian countries. Two studies were reported from Australia.

The studies included in Table I differed in a number of ways in their execution and analysis. Sixteen studies have used as a comparison group or controls subjects selected from defined populations, five selected comparison subjects from hospitals or clinics, and two studies selected comparison subjects from both these sources. Thirteen studies obtained dietary data using food frequency questionnaires, nine with diet histories, and one with a 24 h diet recall. Food frequency questionnaires were sometimes administered by interview and sometimes self administered and it is clear from the accounts given in the publications that the questionnaires differed substantially in the number of food items included (data not shown in Table).

All of the studies included in Table I analysed the relationship between breast cancer risk and nutrient intake by partitioning intake. Five studies partitioned by quintiles, 11 by quartiles, four by tertiles, and one at the media. One study used deciles of intake and one by specified increments in fat intake. Eleven studies met at least six of the methodological standards that were applied, seven met five, and five met four or fewer standards.

### *Estimates of risk for nutrient consumption*

Figure 1 shows the estimates of risk of breast cancer generated by these studies for intake of total fat, and when available, for saturated, monounsaturated and polyunsaturated fat. We also indicate risk estimates that have been adjusted for energy intake and for other risk factors for breast cancer. For total fat, the summary relative risk for all 24 estimates was 1.12 (95% CI 1.04–1.21). Cohort studies had a summary relative risk of 1.01 (95% CI 0.90–1.13) and

**Table I** Characteristics of studies for meta analysis – fat intake

| *Author* | *Country* | *No. of cases* | *No. of controls* | *Type of controls* | *Dietary assessment* | *Partition* | *Relative risk total fat* | *Quality score* |
|---|---|---|---|---|---|---|---|---|
| *Case control:* | | | | | | | | |
| Ewertz (1990) | Denmark | 1,474 | 1,322 | population | food freq[a,b] | quartile | 1.45 (1.17,1.80) | 3/7 |
| Graham (1982) | USA | 1,803 | 917 | hospital | food freq[a,c] | quartile | 0.9 (0.5,1.5) | 5/7 |
| Graham (1991) | USA | 439 | 494 | population | food freq[a,c] | quartile | 0.93 (0.63,1.38) | 5/7 |
| Hirohata (1985) | Japan | 212 | 424 | hospital & neighbourhood combined | diet history[c] | quartile | 1.01 (0.60,1.71) | 3/7 |
| Hirohata Japanese & | Hawaii | J 183 | 183 | neighbourhood | diet history[c] | quartile | 1.5 (0.8,2.9) | 5/7 |
| Caucasian (1987) | | C 161 | 161 | neighbourhood | diet history[c] | quartile | 1.3 (0.6,2.6) | |
| Ingram (1991) | Australia | 99 | 209 | population | food freq[a,b] | median of fat intake | 1.4 (0.8,2.5) | 5/7 |
| Katsouyanni (1988) | Greece | 120 | 120 | hospital | food freq[a] | 90th *vs* 10th percentiles | 1.36 (0.69,2.67) | 4/7 |
| Lee (1991) | Singapore | 200 | 420 | hospital | food freq[a] | tertile | 0.75 (0.41,1.36) | 4/7 |
| Miller (1978) | Canada | 400 | 400 | population | diet history[c] | tertile | 1.6 (0.9,3.0) | 5/7 |
| Pryor (1989) | USA | 172 | 190 | population | food freq[a,c] | quartile | 0.7 (0.3,1.5) | 5/7 |
| Richardson (1991) | France | 409 | 515 | hospital | diet history | tertile | 1.6 (1.1,2.2) | 6/7 |
| Rohan (1988) | Australia | 451 | 451 | population | food freq[a,b,c] | quintile | 0.9 (0.59,1.38) | 6/7 |
| Shun-Zhang (1990) | China | 186 | 372 | population & hospital | diet history[c] | quintile | 1.67 (1.01,2.05) | 6/7 |
| Toniolo (1989) | Italy | 250 | 499 | population | diet history[c] | quartile | 1.8 (0.98,3.29) | 6/7 |
| van't Veer (1990, 1991) | Netherlands | 133 | 289 | population | diet history[c] | per 24 g fat | 1.54 (1.06,2.22) | 6/7 |
| Zaridze (1991) | Moscow | 139 | 139 | clinic | food freq[a] | quartile | 0.52 (0.04,6.99) | 5/7 |
| Total cases: | 6,831 | | | | | | | |
| Total controls: | 7,105 | | | | | | | |
| *Cohort studies:* | | | | | | | | |
| Graham (1992) | USA | 359 | 18,586 | population | food freq[a,b,c] | quintile | 0.99 (0.69,1.41) | 6/6 |
| Howe (1991) | Canada | 519 | 56,837[d] | population | diet history[b,c] | quartile | 1.35 (1.00,1.82) | 6/6 |
| Jones (1987) | USA | 99 | 5,495 | population | 24 h recall | quartile | 0.34 (0.16,0.73) | 3/6 |
| Knekt (1990) | Finland | 54 | 3,988 | population | diet history[c] | tertile | 1.72 (0.61,4.82) | 6/6 |
| Kushi (1992) | USA | 459 | 34,388 | population | food freq[a,b,c] | quartile | 1.16 (0.87,1.55) | 6/6 |
| van den Brandt (1993) | Netherlands | 471 | 62,573[e] | population | food freq[a,b,c] | quintile | 1.08 (0.73,1.59) | 6/6 |
| Willett (1992) | USA | 1,439 | 89,494 | population | food freq[a,b,c] | quintile | 0.90 (0.77,1.07) | 6/6 |
| Total cases: | 3,007 | | | | | | | |
| Total population: | 252,765 | | | | | | | |

[a]Food Frequency Questionnaire. [b]Self administered. [c]Diet assessment method validated. [d]No. of controls in calculation of RR = 1,182. [e]No. of controls in calculation of RR = 1,598.

case control studies a relative risk of 1.21 (95% CI 1.10–1.34). Summary relative risks for both cohort and case control studies were slightly higher when only studies performing adjustment for energy intake and risk factors for breast cancer were included. The estimate for cohort studies was 1.03 (95% CI 0.92–1.16) and for case control studies was 1.42 (95% CI 1.17–1.72). Summary relative risks for saturated fat were greater than unity for all studies combined (RR 1.10; 95% CI 1.00–1.21), case control alone (RR 1.36; 95% CI 1.17–1.58) but not for cohort studies alone (RR 0.95; 95% CI 0.84–1.08); in case control studies that adjusted for energy and risk factors the summary relative risk was 1.31 (95% CI 1.02–1.68). The summary relative risk for monounsaturated fat was 1.09 (95% CI 0.99–1.21) for all studies, 1.42 for case control studies alone (95% CI 1.19–1.69), and 0.95 for cohort studies alone (95% CI 0.84–1.08). Summary relative risks for polyunsaturated fats were consistently one or less but the confidence intervals did not exclude unity in

**Figure 2** Relative risks for **a**, meat **b**, milk and **c**, cheese intake and breast cancer risk in case control and cohort studies. Confidence intervals are 95%. Closed circle = relative risk unadjusted for energy intake or other risk factors; closed square = relative risk adjusted for other risk factors; open square = relative risk adjusted for energy intake and other risk factors. Open circles represent summary relative risk results of the meta-analysis.

any of the analyses carried out (all studies, 0.97; 95% CI 0.88–1.07, case control, 0.92; 95% CI 0.79–1.08, and cohort studies, 1.00; 95% CI 0.89–1.13).

*Characteristics of studies reporting analysis according to foods*

The 19 studies that examined food consumption in relation to breast cancer risk, 14 case control and five cohort in design, included a total of 8,693 cases and over 230,000 control or comparison subjects (Ewert & Gill, 1990; Hirohata *et al.*, 1987; Hislop *et al.*, 1986; Ingram *et al.*, 1991; Kato *et al.*, 1992; La Vecchia *et al.*, 1987; Le *et al.*, 1986; Lee *et al.*, 1991; Lubin *et al.*, 1981; Matos *et al.*, 1991; Richardson *et al.*, 1991; Talamini *et al.*, 1984; Toniolo *et al.*, 1989; Van't Veer *et al.*, 1989; Hirayama, 1984; Kinlen, 1982; Mills *et al.*, 1988; van den Brandt *et al.*, 1993; Vatten *et al.*, 1990). There is some overlap, as seven studies reported risk in relation to consumption of both nutrients and foods and these are also included in Figures 1 and 2. The 19 studies contained 17 estimates of risk for meat, ten for milk and six for cheese.

Table II summarises selected characteristics of the published studies that examined the role of diet in relation to breast cancer risk by an analysis of food intake. Ten studies were carried out in European countries, four in North America, two in Japan, and one each in Argentina, Australia and Singapore.

Thirteen studies used as a comparison group or controls subjects selected from defined populations, and six selected comparison subjects from hospitals. All but five studies obtained dietary data using food frequency questionnaire, and one used unspecified methods.

All of the studies included in Table II analysed the relationship between breast cancer risk and food intake by partitioning intake. Differences in methods of partitioning existed not only between studies but also within studies in analysing intake of different foods. Three studies met at least six of the methodological standards that were applied, two met five, and 14 met four or fewer standards.

*Estimates of risk for food consumption*

Figure 2 shows graphically the distribution of the estimates of risk of breast cancer and the 95% confidence intervals generated by these studies for intake of meat, milk and cheese, the types of food for which data were available from the largest number of studies. The summary relative risks for meat intake were 1.18 (95% CI 1.06–1.32) for all studies,

**Table II** Characteristics of studies for meta analysis – Foods

| *Author* | *Country* | *No. of cases* | *No. of controls* | *Type of controls* | *Dietary assessment* | *Food* | *No of categories*[d] | *Relative risk* | *Confidence interval* | *Quality score* |
|---|---|---|---|---|---|---|---|---|---|---|
| *Case control* | | | | | | | | | | |
| Ewertz (1990) | Denmark | 1,474 | 1,322 | population | food freq[a,b] | meat | 6 | 0.94 | (0.63,1.40) | 3/7 |
| | | | | | | milk | 5 | 1.45 | (1.02,2.07) | |
| Hirohata (1987) | USA | 183 | 183 | population | diet history[c] | meat | 4 | 1.5 | (0.7,3.1) | 5/7 |
| Hislop (1986) | Canada | 846 | 862 | population | food freq[a,b] | meat | 3 | 1.16 | (0.90,1.48) | 3/7 |
| | | | | | | milk | 3 | 1.55 | (1.18,2.05) | |
| Ingram (1991) | Australia | 99 | 209 | population | food freq[a] | meat | 2 | 1.6 | (0.9,2.8) | 3/7 |
| | | | | | | milk | 2 | 0.9 | (0.5,1.6) | |
| Kato (1992) | Japan | 908 | 908 | hospital | unspecified | meat | 3 | 0.75 | (0.60,0.94) | 2/7 |
| La Vecchia (1987) | Italy | 1,108 | 1,281 | hospital | food freq[a] | meat | 3 | 1.39 | (1.12,1.71) | 4/7 |
| Le (1986) | France | 1,010 | 1,950 | hospital | food freq[a] | milk | 3 | 1.8 | (1.3,2.4) | 5/7 |
| | | | | | | cheese | 3 | 1.5 | (1.0,2.3) | |
| Lee (1991) | Singapore | 200 | 420 | hospital | food freq[a] | meat | 3 | 1.4 | (0.77,2.53) | 4/7 |
| Lubin (1981) | Canada | 577 | 826 | population | food freq[a] | meat | 3 | 1.42 | (1.0,2.0) | 4/7 |
| | | | | | | milk | 4 | 0.77 | (0.5,1.3) | |
| | | | | | | cheese | 3 | 1.11 | (0.9,1.4) | |
| Matos (1991) | Argentina | 196 | 205 | neighbourhood | food freq[a] | meat | 3 | 1.4 | (0.7,2.9) | 4/7 |
| Richardson (1991) | France | 409 | 515 | hospital | diet history[a] | meat | 3 | 1.0 | (0.7,1.4) | 6/7 |
| | | | | | | cheese | 3 | 1.4 | (1.0,1.9) | |
| Talamini (1984) | Italy | 368 | 373 | hospital | food freq | meat | 3 | 1.3 | (0.7,2.2) | 4/7 |
| | | | | | | milk | 3 | 3.2 | (1.8,5.8) | |
| Toniolo (1989) | Italy | 250 | 499 | population | diet history[c] | meat | 4 | 1.15 | (0.82,1.62) | 6/7 |
| | | | | | | milk | 4 | 1.73 | (1.16,2.60) | |
| | | | | | | cheese | 4 | 2.6 | (1.7,4.0) | |
| van't Veer (1989) | Netherlands | 133 | 289 | population | diet history | meat | per 285 g | 0.81 | (0.59,1.12) | 4/7 |
| | | | | | | cheese | per 60 g | 0.56 | (0.33,0.95) | |
| Total cases: | 7,761 | | | | | | | | | |
| Total controls: | 9,842 | | | | | | | | | |
| *Cohort studies:* | | | | | | | | | | |
| Hirayama (1978) | Japan | 139 | 142,857 | population | National Nutrition Survey | meat | 2 | 1.7 | (0.8,3.8) | 3/6 |
| Kinlen (1982) | Britain | 62 | 2,813 | population | unspecified | meat | 2 | 1.2 | (0.8,1.6) | 2/6 |
| Mills (1988) | USA | 142 | 16,190[e] | population | food freq | meat | 3 | 1.17 | (0.71,1.94) | 4/7 |
| | | | | | | milk | 4 | 1.03 | (0.56,1.90) | |
| | | | | | | cheese | 4 | 2.6 | (1.7,4.0) | |
| van den Brandt (1993) | Netherlands | 437 | 62,573[f] | population | food freq[a,b,c] | meat | n/a[g] | 1.23 | (0.63,0.37) | 6/6 |
| Vatten (1990) | Norway | 152 | 14,500 | population | food freq[a,c] | meat | 3 | 1.8 | (1.1,3.1) | 4/6 |
| Total cases: | 932 | | | | | | | | | |
| Total controls: | 238,933 | | | | | | | | | |

[a]Food Frequency Questionnaire. [b]Self administered. [c]Diet assessment method validated. [d]No of categories refers to the number of categories of frequency of consumption into which the food intakes were partitioned. The RR is for the highest *vs* lowest level of consumption. [e]No. of controls in calculation of RR = 852. [f]No of controls in calculation of RR = 1,598. [g]Not specified.

1.14 (95% CI 1.02–1.29) for case control studies alone and 1.37 (95% CI 1.07–1.76) for cohort studies alone. The summary relative risk for milk was 1.17 (95% CI 1.04–1.31) and for cheese, 1.17 (95% CI 1.02–1.36). Seven studies provided a risk estimate for red meat alone (i.e. excluded chicken and fish) and the summary relative risk for these red meat estimates was 1.54 (95% CI 1.31–1.82). The summary relative risk for chicken/poultry was 0.94 (95% CI 0.78–1.13; five studies).

*Sensitivity analysis*

As has already been noted, the studies included in this analysis differed in a number of aspects of their design and execution, and were reported from countries that are known to have wide differences in breast cancer risk. We examine below the influence of these sources of heterogeneity on the results presented in the previous sections. Further, we have considered the existence of hypothetical unpublished data, with results that show no relationship between dietary fat and breast cancer risk, and the influence that such data would have on our results.

Because of the small number of studies available after division into subgroups we have confined our attention to those studies that reported the results of nutrient analysis for total fat intake and breast cancer risk.

(1) *Heterogeneity between studies*

*Variation in methodology* The principal sources of variation in study methodology examined were the extent to which studies met the methodological standards described above and the source from which control or comparison groups were selected.

(i) *Methodological standards.* Summary relative risks were calculated for studies classified according to the proportion of methodological standards met (see Methods section). The summary relative risk for the relationship of total fat intake to breast cancer risk, for all 11 studies that met 90% or more standards, was 1.15 (95% CI 1.05–1.27). For the eight studies that met between 70 and 80% of standards, the summary relative risk was 1.06 (95% CI 0.86–1.31), and for the five studies that met 60% or less of the standards the relative risk was 1.07 (95% CI 0.92–1.24).

(ii) *Source of controls.* The summary relative risk for total fat and breast cancer risk was 1.13 (95% CI 1.03–1.23) for the 17 studies in Figure 1 that selected control or comparison groups from defined non-hospital populations. The ten case control studies in this group had a summary relative risk of 1.33 (95% CI 1.16–1.52). The five case control studies that selected controls from hospital populations had a summary relative risk of 0.99 (95% CI 0.83–1.18).

*Partitioning of nutrient intake* The summary relative risk for studies that partitioned nutrient intake in quintiles or quartiles was 1.10 (95% CI 1.01–1.20) for all studies and 1.25 (95% CI 1.10–1.42) for case control studies and for studies that used tertiles 1.03 (95% CI 0.84–1.26) for all studies and 1.01 (95% CI 0.82–1.24) for case control studies.

*Geographical variation* To examine the possible influence of the country in which the studies were carried out, they were divided into three geographical categories: Europe (eight studies), North America (ten studies and 11 estimates of risk) and other (five studies). The summary relative risk for European studies was 1.45 (95% CI 1.26–1.67); for North American studies 1.00 (95% CI 0.90–1.11) and for other areas 1.01 (95% CI 0.85–1.20).

*Regression analysis* To examine the independent contribution of the factors considered above regression analysis was carried out, in which the log of the relative risk for total fat intake in each study, weighted by the reciprocal of its variance, was the dependent variable, and study quality score, energy adjustment, geographical area and study design the independent variables. The result of this analysis showed that geographical area was independently associated with relative risk after taking into account all the other variables. European studies were associated with significantly higher estimates of relative risk than studies performed elsewhere, a difference that persisted when study type (cohort or case control) and quality score were included in the model. There was no evidence of an interaction between quality score and the geographical area in which studies were carried out. As the data in Table II show, European studies included about as many cases as studies from North America, although fewer were of cohort design, and studies done in these two regions were of similar quality.

(2) *Hypothetical unpublished results*

Relative risk estimates were selected randomly with replacement from the pool of 17 null results (case control and cohort) for total fat intake already included in the meta-analysis. These risk estimates were added progressively to the 24 case control and cohort risk estimates included in the actual meta-analysis. One hundred of these simulations indicated that a mean of 17 of these null studies needed to be added to the meta-analysis before the summary relative risk became non significant. When case control studies alone were considered in this analysis, 29 randomly selected null risk estimates had to be added to the meta-analysis of 17 case control studies before the summary risk estimate became non significant.

### Discussion

This quantitative summary of the published literature on the risk of breast cancer associated with dietary fat intake suggests that higher intake of dietary fat is associated with an increased risk of breast cancer. The summary relative risk for all studies that examined nutrient intake is however calculated from the results of cohort and case control studies and the results of these different designs for epidemiological investigation give discrepant results. It is not possible from the information available to explain this discrepancy. Chance effects or one or more of the biases to which case control and cohort studies are liable may explain the discrepancy. For example, bias in selection that affects both exposure and disease risk can distort the results of case control studies. Further, most of the cohort studies reported to date have been carried out in North America, where, as suggested above the range of dietary fat intake may be too narrow to allow the detection of associations with the currently available methods of dietary assessment.

Additional methodological problems concern the validation of methods of dietary measurement and the methods for the adjustment of measured intakes for energy. In interpreting the results of the studies considered here it is obviously important to know to what extent the methods of dietary assessment used measured what they purported to measure. Seventeen of the dietary measurement instruments used in the 23 studies that assessed total fat intake had been examined for evidence of validity. However, there are at present no generally agreed criteria for what constitutes a sufficiently valid method of measurement for use in this context. For example the food frequency questionnaire used in the Nurses Health Study was 'validated' by comparison with food records maintained over a year (Willett *et al.*, 1985). The correlation observed between the two measures was 0.53 and this was apparently judged to be satisfactory, although in other contexts this degree of agreement would be judged at best moderate (Nunnally, 1970). This degree of correlation 'explains' only 25% of the variance in fat intake as described in the food records.

The need to adjust for energy intake is clearly indicated by the results of animal experiments which appear to show effects of both calories and fat intake on mammary tumorigenesis. Further, the results of human studies may be strongly influenced by energy adjustment (see for example Knekt *et al.*, 1990). There is, however, no generally agreed method for performing energy adjustment in the analysis of data relating intake of fat to risk of breast cancer. The study of Kushi *et al.* (1992) illustrates the importance of the choice of method. In using four different methods of energy adjustment applied to the same data Kushi and colleagues showed that point estimates of risk, and the associated statistical tests for trend, may vary, although the 95% confidence intervals around point estimates of risk all overlapped considerably.

This summary of the available evidence suggests that dietary fat and breast cancer risk are associated. This conclusion, derived mainly from case control studies, is supported by the summary relative risks from the 19 studies that examined food intake in relation to breast cancer risk. Among these studies the results of cohort and case control studies were in agreement. These showed meat and dairy products, principal sources of dietary fat in Western populations, to be associated with risk of breast cancer. The summary relative risk of the four cohort studies that examined meat intake and breast cancer risk was similar to the summary relative risk for case control studies.

Three studies measuring total fat intake in breast cancer cases and controls could not be included in the meta-analysis as only mean comparisons of fat intake were reported. In the two case control studies the total fat intake of breast cancer cases was significantly greater than that of the controls (Iscovich *et al.*, 1989; Sarin *et al.*, 1985). The remaining study, a nested case control (Nomura *et al.*, 1978), did not find a significant difference between estimated fat intakes of cases and controls, however, the fat intake estimate used was actually the intake of the husbands of the female breast cancer patients under the assumption that dietary patterns between husbands and wives would be similar. Two studies relating to the intake of foods could not be included in this meta-analysis. Lubin *et al.* (1986) studied the risk of breast cancer associated with consumption of fat containing foods and reported that, in women over the age 50 years, the relative risk comparing the highest and the lowest quartile of intake of fat containing foods was significantly greater than one. However, Iscovich *et al.* (1989) did not find a significantly increased risk of breast cancer associated with meat or cheese intake, and for whole milk consumption the relative risk was significantly less than one. The inclusion of the results of these excluded studies in the meta-analysis would not have weakened the conclusion that the summary of the available evidence indicates that dietary fat intake and breast cancer risk are related.

One of the most important of the biases in the context of the present study is bias in recall, which may cause cases with breast cancer to recall food intake in greater detail or quantity than do healthy controls, creating spurious associations between food intake and disease. Cohort studies, in which information about food intake is collected before the onset of disease, are not susceptible to this form of bias. No evidence for the existence of this bias was reported in one recently reported study (Friedenreich *et al.*, 1991) in which both cohort and case control designs were applied to the same population, although odds ratios did show non-significant differences in the direction predicted by recall bias. Giovannucci *et al.* carried out a case control study in the cohort of the Nurses Health Study and found a non-significant increase, from 0.87 to 1.43, in odds ratios for the association of dietary fat and breast cancer risk (Giovannucci *et al.*, 1991).

In the present study we examined the association of breast cancer risk with specific types of fat and found associations with saturated and monounsaturated fat but not with polyunsaturated fat. For this result to be explained by recall bias it would be necessary to postulate a bias in recall that affects some but not all types of fat.

The findings of this analysis agree with data from animal studies and ecological analysis about the effects of total fat and saturated fat on breast cancer risk. The effects of monounsaturated fats on mammary tumorigenesis have been inconsistent (Welsch, 1992; Pritchard *et al.*, 1989; Cohen *et al.*, 1986), while polyunsaturates of the omega-6 family in general do promote tumorigenesis (Welsch, 1986). Prentice *et al.* (1988) found no association between international breast cancer rates and monounsaturates, but did find an association with polyunsaturates. The data in the papers included in the present analysis were of course obtained from individuals and are expected therefore to more accurately reflect consumption than does the food disappearance data used in ecological analyses.

The present study includes nine of the 12 case control studies re-analysed by Howe *et al.* (1990). Unlike the report of Howe, we were unable to repartition fat intake, but have based our analysis on the partitions selected by the authors of the original reports. Thus the ranges of fat intake for which relative risks were calculated were those observed in the original population studied. One limitation of the approach that we have taken is likely to have attenuated the calculated summary relative risks. If fat intake is indeed related to breast cancer risk, the relative risk generated by a study that partitions fat intake into quintiles will generate a larger relative risk between the highest and lowest categories of intake than does a study that partitions according to tertiles. Evidence consistent with this suggestion is seen in the higher summary relative risk found in studies that used four or more partitions of intake than in those studies that used three or fewer partitions.

Because of differences in methods of partitioning fat intake, as well as differences in the methods of dietary assessment and probable differences in measurement error, we have not attempted to compare the risks observed with those that would be predicted from international variation in fat consumption. The observed summary relative risk from case control studies is however very similar to that predicted from the range of fat intake in the Nurses' Health Study and the measurement error known to be associated with the food frequency questionnaire used in that study (Prentice *et al.*, 1989; Goodwin & Boyd, 1987). The Nurses' Health Study population (Willett *et al.*, 1987), which was confined to a single occupational group, may be more homogeneous in fat consumption than the general population.

Meta-analysis has to date been applied mainly to the results of randomised trials of therapy. Although examples exist of meta-analysis directed at risk factors for disease there is no general agreement on whether this is an appropriate use of the method. Unanswered questions concern whether studies with heterogeneous results should be combined, how differences in study quality should be taken account of, whether studies with hospital based control groups should be combined with those that have population based controls, and whether studies carried out in different countries should be combined (Fleiss & Gross, 1991; Spitzer, 1991). We have addressed several of these issues in this analysis, including the extent to which studies met generally agreed principles of design and analysis for epidemiological research, particularly adjustment for energy intake and risk factors for breast cancer, and the source from which control populations were drawn, as well as the countries in which studies were done. We have also considered the possible influence of hypothetical unpublished studies. None of these considerations weakened the conclusion that fat intake is associated with breast cancer risk, and several strengthened it. Of particular interest is the finding of differences in the results of studies done in Europe and North America which may be due to greater variation in dietary fat intake in Europe, although as dietary measurement instruments differ between studies, the evidence on this point cannot at present be conclusive. We have not however been able to examine the influence of menopausal status on the risk associated with dietary fat because few of the papers examined gave separate estimates of risk for pre and postmenopausal women.

Experimental evidence, derived from controlled clinical trials in which the range of fat intake is increased beyond that seen in most Western populations, would provide the strongest evidence available concerning the relationship of dietary fat intake to breast cancer risk. Further, such trials are the only means likely to answer the question of whether breast cancer risk in high risk subjects can be modified by changing dietary fat intake. The feasibility of an experimental approach to this problem, including the identification of subjects at increased risk for breast cancer, and the demonstration that such subjects will enter a clinical trial of dietary fat reduction and comply with a lower fat diet has been described (Boyd *et al.*, 1990).

This work was supported by grants from the Ontario Ministry of Health, the Medical Research Council of Canada, and by a Terry Fox Programme Project Grant from the National Cancer Institute of Canada. Mr Noffel was supported by a Summer student Scholarship from the Institute of Medical Sciences, University of Toronto. Dr Boyd is the recipient of a National Health Scientist Award, Health and Welfare, Canada.

### References

BAILAR, J.C. & SMITH, E.M. (1986). Progress against cancer? *N. Engl. J. Med.*, **314**, 1226–1232.

BORING, C.C., SQUIRES, T.S. & TONG, T. (1993). Cancer Statistics, 1993. *Ca Cancer J. Clin.*, **43**, 7–26.

BOYD, N.F., COUSINS, M., LOCKWOOD, G. & TRITCHLER, D. (1990). The feasibility of testing experimentally the dietary fat-breast cancer hypothesis. *Br. J. Cancer*, **62**, 878–881.

COHEN, L.A., THOMPSON, D.O., MAEURA, Y., CHOI, K., BLANK, M.E. & ROSE, D.P. (1986). Dietary fat and mammary cancer. I. Promoting effects of different dietary fats on N-nitrosomethylurea-induced rat mammary tumorigenesis. *J. Natl Cancer Inst.*, **77**, 33–42.

DERSIMONIAN, R. & LAIRD, N. (1986). Meta-analysis in clinical trials. *Controlled Clin. Trials.*, **7**, 177–188.

EWERTZ, M. & GILL, C. (1990). Dietary factors and breast-cancer risk in Denmark. *Int. J. Cancer*, **46**, 779–784.

FLEISS, J.L. (1981). *Statistical Methods for Rates and Proportions*. 2nd. Ed. John Wiley & Sons: New York.

FLEISS, J.L. & GROSS, A.J. (1991). Meta-analysis in epidemiology, with special reference to studies of the association between exposure to environmental tobacco smoke and lung cancer: a critique. *J. Clin. Epidemiol.*, **44**, 127–139.

FREEDMAN, L.S., CLIFFORD, C. & MESSINA, M. (1990). Analysis of dietary fat, calories, body weight and the development of mammary tumours in rats and mice: a review. *Cancer Res.*, **50**, 5710–5719.

FRIEDENREICH, C.M., HOWE, G.R. & MILLER, A.B. (1991). The effect of recall bias on the association of calorie-providing nutrients and breast cancer. *Epidemiol.*, **2**, 424–429.

GIOVANNUCCI, E., STAMPFER, M.J., COLDITZ, G.A., MANSON, J., ROSNER, B., LONGNECKER, M., SPEIZER, F.E. & WILLETT, W.C. (1991). A comparison of prospective and retrospective assessments of diet in the study of breast cancer. *Am. J. Epidemiol.*, **134**, 714.

GOODWIN, P. & BOYD, N.F. (1987). Critical appraisal of the evidence that dietary fat intake is related to breast cancer risk in humans. *J. Natl Cancer Inst.*, **79**, 473–485.

GRAHAM, S., HELLMANN, R., MARSHALL, J., FREUDENHEIM, J., VENA, J., SWANSON, M., ZIELEZNY, M., NEMOTO, T., STUBBE, N. & RAIMONDO, T. (1991). Nutritional epidemiology of postmenopausal breast cancer in western New York. *Am. J. Epidemiol.*, **134**, 552–566.

GRAHAM, S., MARSHALL, J., METTLIN, C., RZEPKA, T., NEMOTO, T. & BYERS, T. (1982). Diet in the epidemiology of breast cancer. *Am. J. Epidemiol.*, **116**, 68–75.

GRAHAM, S., ZIELEZNY, M., MARSHALL, J., PRIORE, R., FREUDENHEIM, J., BRASURE, J., HAUGHEY, B., NASCA, P. & ZDEB, M. (1992). Diet in the epidemiology of postmenopausal breast cancer in the New York State cohort. *Am. J. Epidemiol.*, **136**, 1327–1337.

HIRAYAMA, T. (1978). Epidemiology of breast cancer with special reference to the role of diet. *Prev. Med.*, **7**, 173–195.

HIROHATA, T., NOMURA, A.M., HANKIN, J.H., KOLONEL, L.N. & LEE, J. (1987). An epidemiologic study on the association between diet and breast cancer. *J. Natl Cancer Inst.*, **78**, 595–600.

HIROHATA, T., SHIGEMATSU, T., NOMURA, A.M., NOMURA, Y., HORIE, A. & HIROHATA, I. (1985). Occurrence of breast cancer in relation to diet and reproductive history: a case-control study in Fukuoka, Japan. *J. Natl. Cancer Inst. Monogr.*, **69**, 187–190.

HISLOP, T.G., COLDMAN, A.J., ELWOOD, J.M., BRAUER, G. & KAN, L. (1986). Childhood and recent eating patterns and risk of breast cancer. *Cancer Detect. Prev.*, **9**, 47–58.

HOEL, D.G., WAKABAYSHI, T. & PIKE, M.C. (1983). Secular trends in the distributions of the breast cancer risk factors – menarche, first birth, menopause, and weight – in Hiroshima and Nagasaki, Japan. *Am. J. Epidemiol.*, **118**, 78–89.

HOWE, G.R., FRIEDENREICH, C.M., JAIN, M. & MILLER, A.B. (1991). A cohort study of fat intake and risk of breast cancer. *J. Natl Cancer Inst.*, **83**, 336–340.

HOWE, G.R., HIROHATA, T., HISLOP, T.G., ISCOVICH, J.M., YUAN, J.M., KATSOUYANNI, K., LUBIN, F., MARUBINI, E., MODAN, B., ROHAN, T., TONIOLO, P. & SHUNZHANG, Y. (1990). Dietary factors and risk of breast cancer: combined analysis of 12 case-control studies. *J. Natl Cancer Inst.*, **82**, 561–569.

INGRAM, D.M., NOTTAGE, E. & ROBERTS, T. (1991). The role of diet in the development of breast cancer: a case-control study of patients with breast cancer, benign epithelial hyperplasia and fibrocystic disease of the breast. *Br. J. Cancer*, **64**, 187–191.

ISCOVICH, J.M., ISCOVICH, R.B., HOWE, G., SHIBOSKI, S. & KALDOR, J.M. (1989). A case-control study of diet and breast cancer in Argentina. *Int. J Cancer*, **44**, 770–776.

JONES, D.Y., SCHATZKIN, A., GREEN, S.B. & 5 others (1987). Dietary fat and breast cancer in the National Health and Nutrition Examination Survey I Epidemiologic Follow-up Study. *J. Natl Cancer Inst.*, **79**, 465–471.

KATO, I., MIURA, S., KASUMI, F., IWASE, T., TASHIRO, H., FUJITA, Y., KOYAMA, H., IKEDA, T., FUJIWARA, K., SAOTOME, K., ASAISHI, K., ABE, R., NIHEI, M., ISHIDA, T., YOKOE, T., YAMAMOTO, H. & MURATA, M. (1992). A case-control study of breast cancer among Japanese women: with special reference to family history and reproductive and dietary factors. *Breast Cancer Res. Treat.*, **24**, 51–59.

KATSOUYANNI, K., TRICHOPOULOS, D., BOYLE, P. & 5 others. (1986). Diet and breast cancer. A case control study in Greece. *Int. J. Cancer*, **38**, 815–820.

KATSOUYANNI, K., WILLETT, W., TRICHOPOULOS, D., BOYLE, P., TRICHOPOULOU, A., VASILAROS, S., PAPADIAMANTIS, J. & MACMAHON, B. (1988). Risk of breast cancer among Greek women in relation to nutrient intake. *Cancer*, **61**, 181–185.

KINLEN, L.J. (1982). Meat and fat consumption and cancer mortality: a study of strict religious orders in Britain. *Lancet*, **1**, 946–949.

KNEKT, P., ALBANES, D., SEPPANEN, R., AROMAA, A., JARVINEN, R., HYVONEN, L., TEPPO, L. & PUKKALA, E. (1990). Dietary fat and risk of breast cancer. *Am. J. Clin. Nutr.*, **52**, 903–908.

KUSHI, L.H., SELLERS, T.A., POTTER, J.D., NELSON, C.L., MUNGER, R.G., KAYE, S.A. & FOLSOM, A.R. (1992). Dietary fat and post-menopausal breast cancer. *J. Natl Cancer Inst.*, **84**, 1092–1099.

LA VECCHIA, C., DECARLI, A., FRANCESCHI, S., GENTILE, A., NEGRI, E. & PARAZZINI, F. (1987). Dietary factors and the risk of breast cancer. *Nutr. Cancer*, **10**, 205–214.

LE, M.G., MOULTON, L.H., HILL, C. & KRAMAR, A. (1986). Consumption of dairy produce and alcohol in a case-control study of breast cancer. *J. Natl Cancer Inst.*, **77**, 633–636.

LEE, H.P., GOURLEY, L., DUFFY, S.W., ESTEVE, J., LEE, J. & DAY, N.E. (1991). Dietary effects on breast-cancer risk in Singapore. *Lancet*, **337**, 1197–1200.

LUBIN, F., WAX, Y. & MODAN, B. (1986). Role of fat, animal protein, and dietary fiber in breast cancer etiology: a case-control study. *J. Natl Cancer Inst.*, **77**, 605–612.

LUBIN, J.H., BURNS, P.E., BLOT, W.J., ZIEGLER, R.G., LEES, A.W. & FRAUMENI, J.F. Jr (1981). Dietary factors and breast cancer risk. *Int. J. Cancer*, **28**, 685–689.

MARSHALL, J.R., YINSHENG, Q., JUNSHI, C., PARPIA, B. & CAMPBELL, T.C. (1992). Additional ecological evidence: lipids and breast cancer mortality among women aged 55 and over in China. *Eur. J. Cancer*, **28A**, 1720–1727.

MATOS, E.L., THOMAS, D.B., SOBEL, N. & VUOTA, D. (1991). Breast cancer in Argentina: case-control study with special reference to meat eating habits. *Neoplasma*, **38**, 357–366.

MILLER, A.B., KELLY, A., CHOI, N.W. & 7 others. (1978). A study of diet and breast cancer. *Am. J. Epidemiol.*, **107**, 499–509.

MILLS, P.K., ANNEGERS, J.F. & PHILLIPS, R.L. (1988). Animal product consumption and subsequent fatal breast cancer risk among Seventh-Day Adventists. *Am. J. Epidemiol.*, **127**, 440–453.

MOOD, A., GRAYBILL, F. & BOES, D. (1974). *Introduction to the Theory of Statistics*. 3rd Ed. pp. 162. McGraw-Hill: New York.

NOMURA, A., HENDERSON, B.E. & LEE, J. (1978). Breast cancer and diet among the Japanese in Hawaii. *Am. J. Clin. Nutr.*, **31**, 2020–2025.

NUNNALLY, J.C. Jr (1970). *Introduction to Psychological Measurement*. McGraw-Hill: New York.

PRENTICE, R.L., KAKAR, F., HURSTING, S., SHEPPARD, L., KLEIN, R. & KUSHI, L.H. (1988). Aspects of the rationale for the women's health trial. *J. Natl Cancer Inst.*, **80**, 802–814.

PRENTICE, R.L., PEPE, M. & SELF, S.G. (1989). Dietary fat and breast cancer: a quantitative assessment of the epidemiological literature and a discussion of methodologic issues. *Cancer Res.*, **49**, 3147–3156.

PRITCHARD, G.A., JONES, D.L. & MANSEL, R.E. (1989). Lipids in breast carcinogenesis. *Br. J. Surg.*, **76**, 1069–1073.

PRYOR, M., SLATTERY, M.L., ROBISON, L.M. & EGGER, M. (1989). Adolescent diet and breast cancer in Utah. *Cancer Res.*, **49**, 2161–2167.

RICHARDSON, S., GERBER, M. & CENEE, S. (1991). The role of fat, animal protein and some vitamin consumption in breast cancer: a case-control study in southern France. *Int. J. Cancer*, **48**, 1–9.

ROGERS, A.E. & LEE, S.Y. (1986). Chemically induced mammary gland tumors in rats: modulation by dietary fat. In *Dietary Fat and Cancer*, Ip, C., Birt, D.F., Rogers, A.E. & Mettlin, C. (eds). *Progress in Clinical and Biological Research*, **222**, 255–282.

ROHAN, T.E., MCMICHAEL, A.J. & BAGHURST, P.A. (1988). A population-based case-control study of diet and breast cancer in Australia. *Am. J. Epidemiol.*, **128**, 478–489.

SARIN, R., TANDON, R.K., PAUL, S., GANDHI, B.M., KAPUR, B.M.L. & KAPUR, K. (1985). Diet, body fat and plasma lipids in breast cancer. *Indian J. Med. Res.*, **81**, 493–498.

SASAKI, S. & KESTELOOT, H. (1992). Value of Food and Agriculture Organization data on food-balance sheets as a data source for dietary fat intake in epidemiologic studies. *Am. J. Clin. Nutr.*, **56**, 716–723.

SHUN-ZHANG, Y., RUI-FANG, L., DA-DAO, X. & HOWE, G.R. (1990). A case-control study of dietary and nondietary risk factors for breast cancer in Shanghai. *Cancer Res.*, **50**, 5017–5021.

SPITZER, W.O. (1991). Meta-meta-analysis: unanswered questions about aggregating data. *J. Clin. Epidemiol.*, **44**, 103–107.

TALAMINI, R., LA VECCHIA, C., DECARLI, A., FRANCESCHI, S., GRATTONI, E., GRIGOLETTO, E., LIBERATI, A. & TOGNONI, G. (1984). Social factors, diet and breast cancer in a northern Italian population. *Br. J. Cancer*, **49**, 723–729.

TONIOLO, P., RIBOLI, E., PROTTA, F., CHARREL, M. & CAPPA, A.P.M. (1989). Calorie-providing nutrients and risk of breast cancer. *J. Natl Cancer Inst.*, **81**, 278–286.

VAN DEN BRANDT, P.A., VAN'T VEER, P., GOLDBOHM, R.A., DORANT, E., VOLOVICS, A., HERMUS, R.J.J. & STURMANS, F. (1993). A prospective cohort study on dietary fat and the risk of postmenopausal breast cancer. *Cancer Res.*, **53**, 75–82.

VAN'T VEER, P., DEKKER, J.M., LAMERS, J.W.J., KOK, F.J., SCHOUTEN, E.G., BRANTS, H.A.M., STURMANS, F. & HERMUS, R.J.J. (1989). Consumption of fermented milk products and breast cancer: a case-control study in the Netherlands. *Cancer Res.*, **49**, 4020–4023.

VAN'T VEER, P., KOK, F.J., BRANTS, H.A.M., OCKHUIZEN, T., STURMANS, F. & HERMUS, R.J.J. (1990). Dietary fat and the risk of breast cancer. *Int. J. Epidemiol.*, **19**, 12–18.

VAN'T VEER, P., VAN LEER, E.M., RIETDIJK, A., KOK, F.J., SCHOUTEN, E.G., HERMUS, R.J. & STURMANS, F. (1991). Combination of dietary factors in relation to breast cancer occurrence. *Int. J. Cancer*, **47**, 649–653.

VATTEN, L.J., SOLVOLL, K. & LOLKEN, E.B. (1990). Frequency of meat and fish intake and risk of breast cancer in a prospective study of 14,500 Norwegian women. *Int. J. Cancer*, **46**, 12–15.

WELSCH, C.W. (1986). Interrelationships between dietary fat and endocrine processes in mammary gland tumorigenesis. In *Dietary Fat and Cancer*, Ip, C., Birt, D.F., Rogers, A.E. & Mettlin, C. (eds). *Progress in Clinical and Biological Research*, **222**, 623–654.

WELSCH, W.C. (1992). Relationship between dietary fat and experimental mammary tumorigenesis – a review and critique. *Cancer Res.*, **52**, S2040–S2048.

WILLETT, C.W., SAMPSON, L., STAMPFER, M.J., ROSNER, B., BAIN, C., WITSCHI, J., HENNEKENS, C.H. & SPEIZER, F.E. (1985). Reproducibility and validity of a semiquantitative food frequency questionnaire. *Am. J. Epidemiol.*, **122**, 51–65.

WILLETT, W.C., STAMPFER, M.J., COLDITZ, G.A., ROSNER, B.A., HENNEKENS, C.H. & SPEIZER, F.E. (1987). Dietary fat and the risk of breast cancer. *N. Engl. J. Med.*, **316**, 22–28.

WILLETT, W.C., HUNTER, D.J., STAMPFER, M.J., COLDITZ, G., MANSON, J.E., SPIEGELMAN, D., ROSNER, B., HENNEKENS, C.H. & SPEIZER, F.E. (1992). Dietary fat and fiber in relation to risk of breast cancer. An 8-year follow-up. *JAMA*, **268**, 2037–2044.

ZARIDZE, D., LIFANOVA, Y., MAXIMOVITCH, D., DAY, N.E. & DUFFY, S.W. (1991). Diet, alcohol consumption and reproductive factors in a case-control study of breast cancer in Moscow. *Int. J. Cancer*, **48**, 493–501.